# Reply to Letter to the Editor “Challenges in the Implementation of Blood Culture Parameters to Identify Patients at Low Risk of Infective Endocarditis”

**DOI:** 10.1093/ofid/ofaf698

**Published:** 2025-11-27

**Authors:** Nicolas Fourré, Benoit Guery, Matthaios Papadimitriou-Olivgeris

**Affiliations:** Infectious Diseases Service, Lausanne University Hospital and University of Lausanne, Lausanne, Switzerland; Infectious Diseases Service, Lausanne University Hospital and University of Lausanne, Lausanne, Switzerland; Infectious Diseases Service, Lausanne University Hospital and University of Lausanne, Lausanne, Switzerland; Infectious Diseases Service, Hospital of Valais and Institut Central des Hôpitaux, Sion, Switzerland

To the editor—In their insightful commentary, Valsecchi et al [1] raise several important points regarding our validation study [2] of the novel, simplified scoring approach proposed by Freling et al [3] to estimate the risk of infective endocarditis (IE) among patients with Gram-positive coccal bacteremia. This score relies solely on two blood culture-derived variables: the number of positive bottles in the initial blood culture set and persistence of bacteremia for 2 days. Both the number of positive initial blood culture bottles and the persistence of bacteremia have previously been shown to be associated with IE [4–9].

According to the 2023 European Society of Cardiology guidelines, echocardiography is recommended for all patients with bacteremia caused by *Staphylococcus aureus*, *Enterococcus faecalis*, and certain streptococcal species [10]. While this strategy is undoubtedly safe, it places a considerable burden on healthcare resources. The prediction score by Freling et al [3], like all clinical risk scores, was designed with a dual purpose: to minimize the risk of missed IE diagnoses while also limiting unnecessary imaging. Ultimately, however, the usefulness of any diagnostic tool depends on achieving the right balance between diagnostic safety and resource efficiency.

In our study [2], only 2% of IE episodes (12 out of 561) were misclassified as low risk, and the score achieved a negative likelihood ratio of 0.08 (95% CI: 0.05–0.15), which is generally considered strong for ruling out IE. Importantly, this score, like any prediction tool, should never be used in isolation but always in conjunction with clinical judgment.

The main concern raised by Valsecchi et al [1] relates to the score's limited practical applicability. Indeed, it classified the majority of episodes (82%) as high risk, meaning it would not substantially reduce the number of echocardiograms compared with clinical judgment alone. Moreover, some patients categorized as low risk had prosthetic valves, cardiac implantable electronic devices, or embolic events—clinical features that independently warrant echocardiographic evaluation regardless of blood culture characteristics. As Valsecchi et al [1] also noted, persistent bacteremia was uncommon in episodes with only one positive bottle, suggesting that the score's predictive value rests primarily on the number of initially positive bottles.

In conclusion, the simple prediction score proposed by Freling et al [3] effectively identifies bacteremic episodes due to *S. aureus*, streptococci, and *E. faecalis* with a low probability of IE. However, its tendency to classify more than 80% of patients as high risk substantially limits its potential to reduce unnecessary echocardiograms. Furthermore, the inclusion of patients with prosthetic material or embolic events in the low-risk group underscores the need for cautious and selective application of this score in clinical practice.
